# Prognostic Value of LC3B and p62 Expression in Small Intestinal Adenocarcinoma

**DOI:** 10.3390/jcm10225398

**Published:** 2021-11-19

**Authors:** Jeong-Won Kim, Sun-Young Jun, Joon-Mee Kim, Young-Ha Oh, Ghilsuk Yoon, Seung-Mo Hong, Joon-Yong Chung

**Affiliations:** 1 Department of Pathology, Kangnam Sacred Heart Hospital, Hallym University College of Medicine, Seoul 07441, Korea; 2 Department of Pathology, Incheon St Mary’s Hospital, College of Medicine, The Catholic University of Korea, Seoul 21431, Korea; pathssun@gmail.com; 3 Department of Pathology, Inha University College of Medicine, Incheon 22332, Korea; jmkpath@inha.ac.kr; 4 Department of Pathology, Hanyang University College of Medicine, Seoul 04763, Korea; yhoh@hanyang.ac.kr; 5 Department of Pathology, Kyungpook National University School of Medicine, Daegu 41944, Korea; gsyoon@knu.ac.kr; 6 Department of Pathology, Asan Medical Center, University of Ulsan College of Medicine, Seoul 05550, Korea; smhong28@gmail.com; 7 Laboratory of Pathology, National Cancer Institute, Center for Cancer Research, National Institutes of Health, Bethesda, MD 20892, USA; chungjo@mail.nih.gov

**Keywords:** LC3B, p62, prognosis, small intestine, adenocarcinoma

## Abstract

Autophagy, a mechanism that maintains cellular homeostasis, is involved in tumor cell growth and survival in cancer, and autophagy inhibitors have been tested clinical trials for anticancer therapy. To elucidate the clinical and prognostic implications of autophagy in small intestinal adenocarcinoma (SIAC), we assessed the expression of autophagy markers, LC3B and p62, in 171 surgically resected primary SIACs using automated quantitative analysis. Positive LC3B, p62 nuclear (p62^Nu^), and p62 cytoplasmic (p62^Cy^) expression was observed in 23 (13.5%), 52 (30.4%), and 43 (25.1%) carcinomas, respectively. LC3B+ expression was correlated with undifferentiated carcinoma (*p* < 0.001) and high histologic grade (*p* = 0.029). The combined expression of LC3B and p62^Nu^ (LC3+/p62^Nu^+) was related to the older age of patients (*p* = 0.017), undifferentiated carcinoma (*p* < 0.001), and high grade (*p* = 0.031). LC3B+ (*p* = 0.006), p62^Cy^+ (*p* = 0.041), or p62^Nu^+ (*p* = 0.006) expression were associated with worse survival. In addition, SIAC patients with either LC3B+/p62^Nu^+ (*p* = 0.001) or LC3B+/p62^Cy^+ (*p* = 0.002) expression had shorter survival times. In multivariate analysis, LC3B expression remained an independent prognostic factor (*p* = 0.025) for overall survival. In conclusion, autophagy may play a role in the tumorigenesis of SIACs, and LC3B and p62 could be used as prognostic biomarkers and potential therapeutic targets for SIACs.

## 1. Introduction

Small bowel cancer is rare in Korea, with 976 new cases occurring in 2018 [1]. However, its incidence is increasing; it reached 1.9 per 100,000 in 2018 compared to 0.9 per 100,000 in 2003 [2]. The proportion of small intestinal cancers among gastrointestinal (GI) malignancies has also increased from 0.69% in 2003 to 1.06% in 2018 [1,2]. Small intestinal adenocarcinoma (SIAC) is the most common histologic type of cancer originating in the small bowel, which accounts for an estimated 30% to 40% of small intestinal cancer diagnoses [3]. Although the mucosal surface of the small bowel covers >90% of the digestive canal, the overall prevalence of SIAC among carcinomas of the tubular GI tract is <2% [1]. This prompts the hypothesis of a protective environment associated with enzymes specific to the small bowel; however, it may be related to the shorter transit time of dietary carcinogens [4]. The small intestine is long and heterogeneous, leading to the development of adenocarcinomas at different sites, with different etiologies and diverse histologic and molecular subtypes [5,6,7]. Consequently, the mechanisms underlying the development and progression of SIAC remain poorly understood. In addition, more than 30% of SIAC patients present with stage IV disease, and, on average, the outcomes of SIAC are worse than those of other GI tract malignancies, including colorectal cancer [7,8]. Therefore, more reliable novel biomarkers, which can be early prognostic indicators and potential treatment targets, are necessary.

Autophagy is a lysosomal pathway that is essential for survival, differentiation, development, and cellular homeostasis by maintaining energy homeostasis, eliminating defective organelles and proteins, preventing reactive oxygen species, and removing intracellular pathogens [9]. It is initiated by the progressive segregation of cytoplasmic material via the phagophore. After fusing with lysosomes to form autolysosomes, the content is degraded by catalytic enzymes, and the products of these reactions are recycled by anabolic or bioenergetic circuitries [10]. Dysfunction of autophagy is associated with cancer as well as autoimmune, cardiac, and neurodegenerative diseases [9]. Under normal conditions, autophagy is tumor suppressive due to its catabolic potential. However, during cancer development, autophagy aids in overcoming stressful stimuli, such as hypoxia and nutrient deprivation. Subsequently, autophagy supports cancer cell growth and facilitates malignant progression in established tumors [11]. Moreover, autophagy-deficient tumors are more sensitive to chemotherapeutic agents and radiation therapy than their autophagy-proficient counterparts [12]. Hence, autophagy is an attractive target for cancer therapeutics, and researchers have been exploiting the use of autophagy modulators as adjuvant therapy [13].

The autophagic process is regulated by a series of autophagy-related genes (*ATGs*) [14]. Among known *ATGs*-encoded proteins, lipidated microtubule-associated protein 1 light chain 3 B (LC3B) is incorporated into the inner and outer autophagosomal membrane, thereby allowing the detection of different stages of autophagic vesicles [15,16]. The incorporated LC3B binds to the adaptor protein p62/sequestosome 1 (SQSTM1), which facilitates autophagic degradation of ubiquitinated protein aggregates in lysosomes [17]. During autophagic flux, both proteins are degraded into autolysosomes [18,19]. Based on these properties, LC3B and p62 have been widely used as biomarkers to monitor autophagy [16]. Immunodetection of autophagy markers has been reported in various human cancers [20,21,22,23,24,25,26]. Aberrant expression of LC3B has been reported in malignant tumors, including pancreatic [20], hepatocellular [21], and colorectal [22,23] carcinomas. Similarly, abnormal expression and regulation of p62 are closely associated with the development and progression of several tumors, such as endometrial [24], breast [25], and colon [26] cancers. However, the clinical and prognostic value of LC3B and p62 expression in patients with SIAC have not yet been studied. The aim of this study was to explore the clinical significance of autophagy-related proteins, including LC3B and p62, for patients with SIAC using immunohistochemistry (IHC) and quantitative digital image analysis.

## 2. Methods

### 2.1. Study Population

We used human SIAC tissue microarrays (TMAs), as previously reported [27,28]. The TMAs were from 197 surgically resected primary SIAC cases collected from the surgical pathology archives of 22 Korean institutions by the Korean Small Intestinal Cancer Study Group [27,28]. The tumor was considered a primary SIAC when the tumor was solitary or predominantly involved the mucosa of duodenum, jejunum and ileum regardless of extension into the serosa, without considering the presence of peritumoral dysplasia. Carcinomas extending from the surrounding gastrointestinal tract organs, such as the stomach, ampulla of Vater, pancreas, cecum or appendix, into the small bowel were excluded. Neuroendocrine tumors and mesenchymal tumors arising in small intestine was also not included. Clinical and pathologic data collected and analyzed in previous studies were used [27,28]. Cancer stages were determined according to the American Joint Committee for Cancer (AJCC), eighth edition [29]. Histological subtypes and grades were classified according to the 2019 World Health Organization (WHO) classification [30]. Information on predisposing conditions, such as Crohn’s disease, familial adenomatous polyposis (FAP), Lynch syndrome, Peutz-Jeghers syndrome, Gardner syndrome, gluten-sensitive enteropathy, intestinal duplication, Meckel’s diverticulum, or heterotopic pancreas was obtained through a review of medical records. This retrospective study was approved by the Institutional Review Board of Incheon St. Mary’s Hospital (OC13SISI0162), and informed consent was waived because the study used leftover specimens. All procedures were conducted in accordance with the principles of the Declaration of Helsinki.

### 2.2. IHC

TMAs were constructed as described previously [31]. They included three invasive adenocarcinoma tissue cores of one-millimeter diameter per patient and one corresponding normal small intestinal mucosa tissue core.

Tissue specimens were cut and used on 5 μm thick paraffin sections. After deparaffinization and rehydration, heat-induced antigen retrieval was performed for 20 min by incubating the samples in antigen retrieval buffer pH 9.0 (DAKO, Carpinteria, CA, USA) for LC3B and pH 6.0 (DAKO) for p62 using a steam pressure cooker (Pascal; DAKO). Endogenous peroxidase activity was quenched with 3% hydrogen peroxide (H_2_O_2_) for 10 min. The sections were incubated with rabbit polyclonal anti-LC3B (Cat no., ab48394; 1:2000; Abcam, Cambridge, MA, USA) and mouse monoclonal anti-p62 (clone D5L7G; 1:1500; Cell Signaling Technology, Danvers, MA, USA) for 1 h at room temperature. Antigen-antibody reactions were detected with EnVision^+^ Dual-HRP (DAKO) and visualized with 3,3-diaminobenzadine (DAB; DAKO). Peripheral nerve tissue with ganglion cells served as an internal positive control for LC3B [16]. Incubation with immunoglobulin G (IgG) or without the primary antibody was performed to generate the negative controls. Finally, the stained sections were lightly counterstained with hematoxylin.

### 2.3. Evaluation of IHC

Stained TMA slides were scanned using a NanoZomer XR Digital Pathology (NDP) system (Hamamatsu, Hamamatsu, Japan) at × 40 objective magnification with a single-focus layer. The tissue on the slides was automatically detected with focus points to obtain the optimal image. Digitalized images were automatically analyzed using Visiopharm software v6.9.1 (Visiopharm, Hørsholm, Denmark) as previously described [32]. In brief, a pathologist (JWK) blinded to patient outcome and other clinical findings generated screenshots of single representative areas of the regions of interest. Blue-colored (hematoxylin) tumor cell nuclei were initially defined, and then brown-colored (DAB) nuclei and cytoplasm were separated spectrally. The brown cytoplasmic intensity (weak and strong) of LC3B and p62 was obtained, and each proportion was analyzed using a predefined algorithm and optimized settings. For p62, nuclear staining was also evaluated. The brown nuclear staining intensity (0 = negative, 1 = weak, 2 = moderate, and 3 = strong) and the percentages of stained cells were obtained, and histoscores were calculated by multiplying the percentage of positive cells with their staining intensity. The average of the three tumor cores was calculated as the final value. Expression values for cytoplasmic staining and histoscores were dichotomized (negative vs. positive), with cutoff values showing the most discriminative power. The cutoff value of LC3B and p62 cytoplasmic expression was set at 1.8% with a strong punctate pattern and 68.6% with weak intensity, respectively. The cutoff histoscore for p62 nuclear expression was 112.6.

### 2.4. Microsatellite Instability (MSI) Analysis

MSI data were obtained from a previous study in the same cohort [28]. Briefly, the five microsatellite loci (BAT25, BAT26, NR21, NR24, and NR27) were amplified in a single multiplex polymerase chain (PCR) reaction. PCR products were analyzed by capillary electrophoresis using an ABI 310 Genetic Analyzer (Applied Biosystems, Foster City, CA, USA). High-frequency MSI (MSI-H) was defined as the presence of two or more loci showing instability, whereas instability at only one locus was defined as low-frequency MSI (MSI-L). Tumors with no instability were defined as microsatellite stable (MSS).

### 2.5. Statistical Analysis

Categorical data were assessed using χ^2^ or Fisher’s exact tests and Mann–Whitney or unpaired Student’s t test, and correlation analyses were applied to compare continuous variables. All survival analyses used an overall survival (OS) model, which captured all patient deaths as events and censored other patients at their last visit dates. OS curves with log-rank tests were generated using the Kaplan–Meier method. Univariate and multivariate survival analyses were performed using the Cox proportional hazard regression model. The statistical significance was set at *p* < 0.05. The SPSS Statistics for Windows, version 21 (IBM Corp., Armonk, NY, USA) was used for the analyses.

## 3. Results

### 3.1. Study Population and Tumor Characteristics

**Figure 1:**
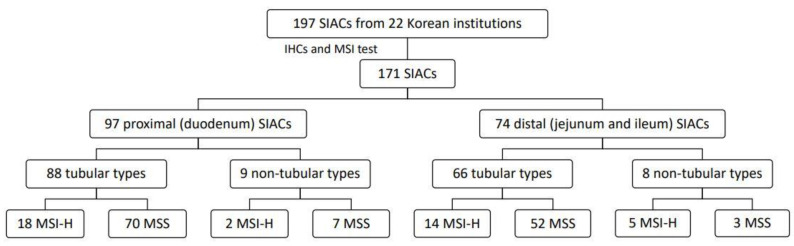
Flow chart of study population and tumor characteristics. SIAC, small intestinal adenocarcinoma; IHC, immunohistochemistry; MSI, microsatellite instability—high; MSS, microsatellite stable.

Out of the enrolled 197 primary SIACs, 171 (86.8%) with interpretable immunohistochemical and molecular results were analyzed in this study (Table 1). The median age of the patients was 59 years (range, 23–86 years), and they were predominantly men (male:female = 1.6:1). There were 97 (56.7%) duodenal, 48 jejunal (28.1%), and 26 ileal (15.2%) cancers. Histologically, 154 patients (90.1%) were tubular adenocarcinomas: 28 tumors (16.4%) were well differentiated, 94 (54.9%) were moderately differentiated and 32 (18.7%) were poorly differentiated. The histologic grade was categorized as low grade (well and moderately differentiated) in 131 (76.6%) and high grade (poorly differentiated and undifferentiated) in 40 (23.4%) tumors. Twenty-four patients (14.0%) had predisposing diseases: 11 patients had adenoma, 7 patients had Lynch syndrome, 1 patient had adenoma and Lynch syndrome, 2 patients had congenital anomaly, 1 patient had Crohn’s disease, and 2 patients had Peutz-Jeghers syndrome. MSI was observed in 39 cases (22.8%), all of which were MSI-H. Figure 1 shows MSI status and tumor characteristics. Sixty-three (38.0%) and 89 (53.5%) tumors were classified as stage II and III, respectively. The median follow-up period after surgical resection was 28.4 months (range, 0.34 to 168.4 months).

### 3.2. IHC Expression of LC3B and p62

**Figure 2:**
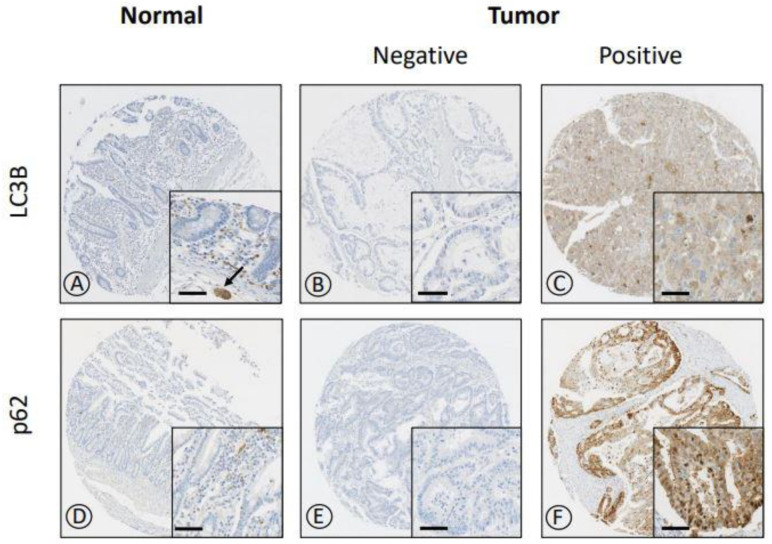
Representative immunohistochemical staining for LC3B and p62 expression in SIAC and normal mucosal tissue. Normal small intestinal epithelial cells did not express LC3B and p62. Some stromal mononuclear cells were weakly reactive for LC3B and p62 and showed punctate or linear cytoplasmic staining ((**A**,**D**), inset). Ganglion cells showed positive staining for LC3B and thus served as an internal staining control (black arrow, (A), inset). LC3B staining showed a cytoplasmic punctate pattern (**C**), while p62 showed cytoplasmic punctate pattern with/without nuclear expression (**F**). The middle columns show representative negative stainings (**B**,**E**) (original magnification, ×8; inset, ×40; scale bar, 50 μm).

**Figure 3:**
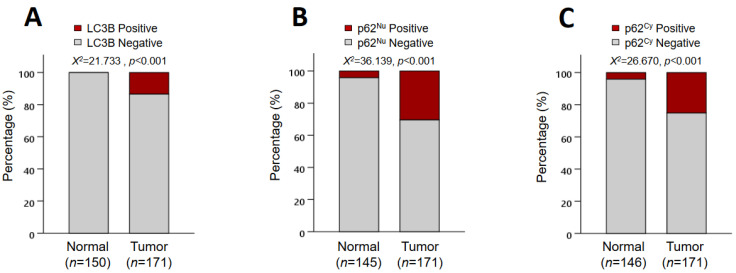
LC3B and p62 expression in SIAC and non-neoplastic small intestinal epithelial cells. LC3B was expressed in malignant tumor cells but not in non-neoplastic epithelial cells (*p* < 0.001) (**A**). P62^Nu^+ (**B**) and p62^Cy^+ (**C**) expressions were more frequently observed in carcinoma than in non-neoplastic epithelium (both *p* < 0.001).

Representative immunohistochemical images of LC3B and p62 are shown in Figure 2. LC3B showed a cytoplasmic punctate expression pattern. Meanwhile, p62 was expressed in both the nucleus (p62^Nu^) and cytoplasm (p62^Cy^) with or without a punctate pattern. All non-neoplastic mucosae of the small intestine were negative for LC3B, and 95.9% of them were negative for p62 (Figure 3). Of the 171 SIACs, positive LC3B (LC3B+) expression was observed in 23 patients (13.5%), whereas p62^Cy^+ and p62^Nu^+ expression was noted in 43 (25.1%) and 52 (30.4%) cases, respectively. LC3B+, p62^Nu^+, and p62^Cy^+ expression was significantly higher in SIACs than in normal mucosae of the small intestine (χ^2^ test; all *p* < 0.001) (Figure 3).

The relationship between LC3B and p62 expression patterns is shown in Table 2. Positive p62^Nu^ expression was strongly associated with p62^Cy^+ expression (*p* < 0.001). LC3B+ expression was significantly correlated with p62^Cy^+ expression (*p* = 0.003); however, there was no relationship between LC3B+ and p62^Nu^+ expression. Twelve (7.0%) cases showed positive cytoplasmic staining for both LC3B and p62 (LC3B+/p62^Cy^+) and 11 (6.4%) cases showed both LC3B cytoplasmic and p62 nuclear positivity (LC3B+/p62^Nu^+).

### 3.3. Association between Clinicopathologic Features and p62 and LC3B Expression

The relationships between the clinicopathological features of SIACs and LC3B and p62 expression are summarized in Table 3. LC3B+ expression was significantly associated with undifferentiated carcinoma (*p* < 0.001) and high histologic grade (*p* = 0.029). Positive p62^Cy^ expression tended to be associated with undifferentiated carcinoma (*p* = 0.056) and observed in patients aged ≥60 years (*p* = 0.056). Tumors with predisposing conditions showed frequent p62^Nu^+ (*p* = 0.044) and p62^Cy^+ (*p* = 0.023) expression. No association was observed between LC3B and p62 expression and other clinicopathological variables including gender, growth type, tumor location and stage, Lynch syndrome (not shown), and MSI status.

The relationship between the combined expression of LC3B and p62 and clinicopathological variables was also examined (Table 4). LC3B+/p62^Nu^+ expression was correlated with older age (*p* = 0.017), undifferentiated histologic type (*p* = 0.031), and higher histologic grade (*p* < 0.001). LC3B+/p62^Cy^+ expression was associated with an undifferentiated histology (*p* < 0.001). SIACs with age ≥60 years and high grade showed frequent LC3B+/p62^Cy^+ expression; however, the difference did not reach statistical significance (*p* = 0.056 and 0.057, respectively).

### 3.4. Prognostic Significance of LC3B and p62 Expression

**Figure 4:**
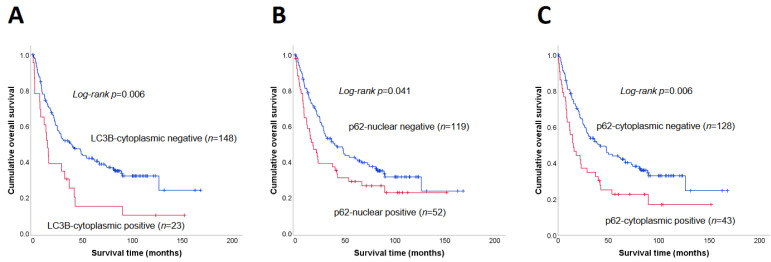
The Kaplan–Meier OS analysis of patients with SIAC. Patients with LC3B+ (**A**), p62^Nu^+ (**B**), and p62^Cy^+ (**C**) expression showed poor OS compared to patients with negative expression for each of these proteins (log rank *p* = 0.006, 0.041, and 0.001, respectively).

**Figure 5:**
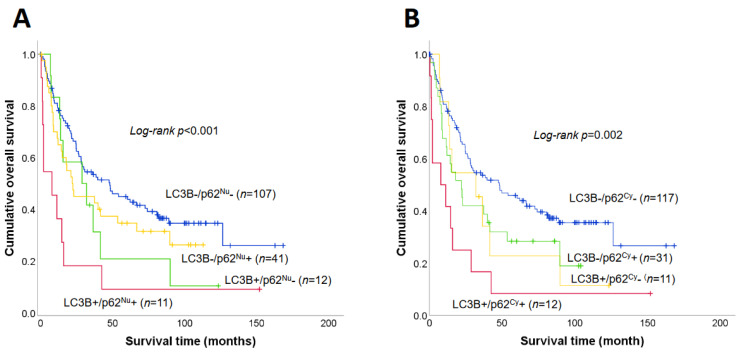
Survival analysis of SIAC patients based on the combination of LC3B and p63 expression. Survival differences were observed among four SIAC patient groups classified according to their LC3B and p62^Nu^ expression and LC3B and p62^Cy^ expression. The survival time of patients with LC3B+/p62^Nu^+ expression was significantly shorter (median 7.9 months) than those with LC3B−/p62^Nu^− (median 48.1 months), LC3B−/p62^Nu^+ (median 22.0 months), and LC3B+/p62^Nu^− (median 28.8 months) (log rank *p* < 0.001) (**A**). Patients with LC3B+/p62^Cy^+ expression had significantly shorter OS (median 7.9 months) than those with LC3B−/p62^Cy^− (median 48.1 months), LC3B−/p62^Cy^+ (median 22.0 months), and LC3B+/p62^Cy^− (median 32.0 months) (log rank *p* = 0.002) (**B**).

The median survival time of patients with SIAC with LC3B+ expression (14.7 months) was shorter than that of patients with LC3B− (38.5 months; *p* = 0.006, log-rank test) (Figure 4A). Patients with each p62^Nu^+ (median survival, 17.8 months) and p62^Cy^+ (15.1 months) expression had notably shorter survival times than patients with p62^Nu^− (39.7 months; *p* = 0.041) and p62^Cy^− (41.6 months; *p* = 0.006) expression, respectively (Figure 4B,C). The survival differences were compared between patients with different combinations of expression patterns of the autophagy markers (Figure 5). The median survival times of patients with LC3B+/p62^Nu^+ (*n* = 11), LC3B+/p62^Nu^− (*n* = 12), LC3B−/p62^Nu^+ (*n* = 41), and LC3B−/p62^Nu^− (*n* = 107) expression were 7.9, 22.0, 28.8, and 48.1 months, respectively. There was a significant survival difference among the four groups (*p* < 0.001, log-rank test, overall comparison) (Figure 5A). Patients with LC3B+/p62^Cy^+ expression (*n* = 12) also had shorter median survival times (7.9 months) than those with LC3B+/p62^Cy^− (*n* = 11; 32.0 months), LC3B−/p62^Cy^+ (*n* = 31; 22.0 months), and LC3B−/p62^Cy^− (*n* = 117; 48.1 months) expression *(p* = 0.002, overall comparison) (Figure 5B).

The relationships between other clinicopathological variables and survival are summarized in Table 5. Univariate analysis revealed that shorter patient survival was associated with undifferentiated histologic type (*p* = 0.008), lymphovascular invasion (*p* < 0.001), MSS (*p* = 0.029), pT classification (*p* = 0.025), lymph node metastasis (*p* < 0.001), and stage (*p* = 0.001).

Multivariate analysis indicated that MSS (*p* = 0.004), higher T stage (*p* = 0.005), lymph node metastasis (*p* = 0.013), and LC3B+ expression (*p* = 0.025) were independent prognostic factors (Table 6). The hazard ratio for SIAC with LC3B+ expression was 1.817 (95% confidence interval, 1.077–3.064) compared to that of LC3B− expression.

## 4. Discussion

The present study highlights the prognostic impact of autophagy-related markers, LC3B and p62, in SIACs. First, we described the immunohistochemical expression patterns of LC3B and p62 proteins in our cohort of SIACs. There are various cellular assays for assessing autophagy, such as transmission electron microscopy, western blotting, flow cytometry, and fluorescence microscopy [18]. In formalin-fixed paraffin-embedded human tissue, IHC is a valuable modality that may provide information about the static level of autophagy-related proteins and prove to be useful in identifying patients for targeted therapy for modulating autophagy in clinical applications [16,18]. Considering the roles of LC3B and p62 in the formation of autophagosomes, overexpression of LC3B and p62 may be observed mainly after the activation of autophagy, but they do not necessarily indicate high levels of active, ongoing autophagy [16,33]. The inhibition of autophagy may prevent the degradation of autophagosomes and cause their accumulation. Therefore, high levels of LC3B and p62 may reflect a defective autophagy pathway [16].

We observed high levels of LC3B and p62 selectively in cancer cells, as previously described for colon and gastric cancers [33,34]. With regard to staining pattern, LC3B exhibited nuclear staining, whereas p62 showed definite nuclear expression in addition to cytoplasmic staining with punctate or diffuse patterns [26,35]. The significance of p62^Cy^ and p62^Nu^ expression for the assessment of autophagy remains unclear [16]. However, both staining patterns have been interpreted as surrogates of autophagy [36,37,38,39]. We found a strong association between p62^Cy^+ and p62^Nu^+ expression (*p* < 0.001, Table 2). In addition, the level of p62^Cy^ expression positively correlated with the level of p62^Nu^ expression (Pearson correlation coefficient, 0.922; *p* < 0.001). Thus, the significance of the p62^Nu^ staining pattern may be comparable to that of p62^Cy^ staining. Nucleocytoplasmic shuttling of p62 has been reported in an in vivo study [40]. In addition, LC3B+ expression was related to p62^Cy^+ expression (*p* = 0.003, Table 2) and the level of LC3B expression was correlated with each the levels of p62^Cy^ or the levels of p62^Nu^ expression (correlation coefficients, 0.240 and 0.304; *p* = 0.002 and *p* < 0.001, respectively). Hence, we conclude that p62 protein, in combination with LC3B, might be used as an ancillary marker of autophagy regardless of the staining pattern. The close relationship between LC3B, p62^Cy^, and p62^Nu^ expression has been described in gastric cancer [33].

Our data demonstrated that autophagy marker expression was associated with the aggressive behavior of SIACs. LC3B+, p62^Nu^+, or p62^Cy^+ expression was associated with shorter survival of patients with SIAC. Moreover, LC3B expression was found to be an independent prognostic factor in the multivariate analysis. Similar to our results, in previous studies on gastric [33] and colorectal [39] cancers, LC3B expression was related to worse prognosis. However, it has been reported that LC3B expression is inversely correlated with poor prognosis in esophageal [37] and colon [34] cancers. The discrepancies can be attributed to various factors, such as organ specificity, characteristics of the tumor itself, genetic factors, antibody clone used in the study, IHC conditions, and cutoff for expression. In our study, strong cytoplasmic staining with a punctate pattern was regarded as positive LC3B staining, and many researchers suggested that only punctate staining pattern correlated with autophagy induction and poor prognosis as opposed to diffuse cytoplasmic staining [26,34].

The prognostic impact of p62 expression in GI adenocarcinomas is also controversial [33,34,39,41]. Yoon et al. [41] and Masuda et al. [33] reported that high p62^Nu^ and p62^Cy^ expression were associated with worse OS in gastric adenocarcinoma. On the contrary, Schmitz et al. [39] and Niklaus et al. [34] showed that high p62^Cy^ expression was significantly correlated with favorable OS in colorectal cancer, while p62^Nu^ expression level was not associated with OS. Regarding the combination of LC3B and p62 staining, we found that SIAC patients with either LC3B+/p62^Nu^+ or LC3B+/p62^Cy^+ expression had shorter survival times than those with other combinations of IHC phenotypes. However, Niklaus et al. showed that tumors with high LC3B/high p62^Cy^ expression had the best OS, whereas tumors with high LC3B/low p62^Cy^ expression showed the worst outcome [34]. This inconsistency might be due to the wide range of functions of p62, including those in the autophagy pathway, the regulation of cell death, and the activation of transcription factor NF-kB [18]. Furthermore, the levels of p62 can be transcriptionally regulated by non-autophagic stimuli, such as the mitogen-activated protein kinase (MAPK) signaling pathway [18] and some p62 positive structures might not reflect autophagosomes. We observed more frequent p62 expression (25.1% for p62^Cy^+ and 30.4% for p62^Nu^+) than LC3B expression (13.5%) in SIACs.

Autophagy was previously thought to play a protective role against malignant transformation. Recently, the role of autophagy in cancer progression and resistance to therapy has gained increased attention. Sakanshi et al. showed that LC3B was significantly associated with high pT category and lymphatic and perineural invasion in colorectal cancers [22]. Masuda et al. reported that age ≥60 years, intestinal type, and lymphatic and vascular invasion were positively related to the expression of autophagy markers in gastric cancers [33]. In addition, it has been reported that autophagy promotes the progression of cancer of the upper GI tract at an early clinical stage [33,42]. We found that either LC3B+ or LC3B+/p62^Nu^+ expression was correlated with the undifferentiated type and high histologic grade. LC3B+/p62^Nu^+ expression was also more frequently observed in SIAC patients aged ≥60 years. However, subgroup analysis by stage did not reveal an association between stage and the expression of autophagy markers in our study cohort. Sena et al. showed that the expression of LC3B in MSS colorectal cancer cells was higher than that in MSI cancer cells [43]. However, we did not identify any differences in the expression of autophagy-related proteins between MSI- and MSS-SIAC patients. Lynch syndrome was also unrelated to the expression of autophagy markers in SIACs, although p62^Cy^+ and p62^Nu^+ expression was frequent in cases with predisposing conditions.

A limitation of this study is that patients with stage IV disease were not included because only surgically resected SIAC specimens were collected. In addition, Crohn’s disease is a well-known predisposing factor for SIACs in the Western population, but rarely in Korean patients [32]. Indeed, only one (0.6%) Crohn’s disease-associated SIAC was observed in our study cohort. Recent advances in genetics have revealed that polymorphisms in autophagy-related 16-like 1 (*ATG16L1*) gene, which is essential for LC3 lipidation and autophagosome formation, are a genetic risk factor for Crohn’s disease [44]. Hence, further studies in larger cohorts might provide insight into the roles of autophagy in the carcinogenesis of SIACs with heterogeneous clinical characteristics.

In conclusion, the high levels of expression of LC3B and p62 proteins selectively in tumor cells of SIACs suggests that the autophagic process is related to tumorigenesis. The correlation between LC3B, p62^Nu^, and p62^Cy^ expression indicates that p62 protein is a surrogate marker of autophagy, irrespective of the staining pattern. Of note, we observed that LC3B and p62 expression, as well as the combined expression of LC3B and p62, have an impact on cancer progression and are related to patient survival. Moreover, LC3B was frequently expressed in tumors with an undifferentiated type and a higher histologic grade. Therefore, LC3B and p62 are potential prognostic biomarkers and promising candidate targets for the treatment of SIACs. Further investigation into the detailed mechanism of LC3B and p62 regulation may provide the basis for anti-cancer therapy through autophagy modulation in SIACs.

## Figures and Tables

**Table 1:** Clinicopathologic characteristics of 171 patients with SIACs.

Category	N	%
Age		
<60 years	95	55.6
≥60 years	76	44.4
Sex		
Male	106	62.0
Female	65	38.0
Location		
Proximal (duodenum)	97	56.7
Distal (jejunum and ileum)	74	43.3
Type of growth ^a^		
Polypoid	31	19.0
Flat	11	6.8
Ulceroinfiltrative	121	74.2
Histological subtype		
Adenocarcinoma	154	90.1
Mucinous carcinoma	9	5.3
Signet ring cell carcinoma	4	2.3
Undifferentiated carcinoma	4	2.3
Tumor grade		
Low	131	76.6
High	40	23.4
Lymphovascular invasion		
Absent	78	45.6
Present	93	54.4
Predisposing condition		
Absent	147	86.0
Present	24	14.0
MSI status		
MSS	132	77.2
MSI-H	39	22.8
pT classification		
pT_is_ − pT_1_	9	5.3
pT_2_	8	4.7
pT_3_	58	33.9
pT_4_	96	56.1
pN classification ^b^		
pN_0_	77	46.4
pN_1_ + pN_2_	89	53.6
AJCC staging ^c^		
0–I	14	8.4
II	63	38.0
III	89	53.6
Status		
Alive	58	33.9
Expire	113	66.1

^a^ Calculated only 163 cases with available information of growth type. ^b^ Calculated only 166 cases with available information of lymph node metastasis. ^c^ Calculated only 166 cases with available information of the AJCC stage.

**Table 2:** Relationship among the expression patterns of LC3B and p62.

	LC3B, *N* (%)			p62^Nu^, *N* (%)		
	Negative	Positive	*p*-Value	Negative	Positive	*p*-Value
p62^Cy^			0.003 *			<0.001 *
Negative	117 (79.1)	11 (47.8)		118 (99.2)	10 (19.2)	
Positive	31 (20.9)	12 (52.2)		1 (0.8)	42 (80.8)	
p62^Nu^			0.088			
Negative	107 (72.3)	12 (52.2)				
Positive	41 (27.7)	11 (47.8)				

* Statistically significant (*p* < 0.05).

**Table 3:** The association between clinicopathologic features and p62 and LC3B expression in patients with SIAC.

	LC3B, *N* (%)			p62^Nu^, *N* (%)			p62^Cy^, *N* (%)		
	Negative *N* = 148	Positive *N* = 23	*p*-Value	Negative *N* = 119	Positive *N* = 52	*p*-Value	Negative *N* = 128	Positive *N* = 43	*p*-Value
Gender			0.300			0.438			0.503
Male	89 (60.1)	17 (73.9)		71 (59.7)	35 (67.3)		77 (60.2)	29 (67.4)	
Female	59 (39.9)	6 (26.1)		48 (40.3)	17 (32.7)		51 (39.8)	14 (32.6)	
Age			0.139			0.071			0.056
<60 years	86 (58.1)	9 (39.1)		72 (60.5)	23 (44.2)		77 (60.2)	18 (41.9)	
≥60 years	62 (41.9)	14 (60.9)		47 (39.5)	29 (55.8)		51 (39.8)	25 (58.1)	
Tumor location			0.805			1.000			0.693
Proximal (duodenum)	85 (57.4)	12 (52.2)		68 (57.1)	29 (55.8)		71 (55.5)	26 (60.5)	
Distal (jejunum and ileum)	63 (42.6)	11 (47.8)		51 (42.9)	23 (44.2)		57 (44.5)	17 (39.5)	
Histological subtype			<0.001 *			0.068			0.056
Adenocarcinoma	136 (91.9)	18 (78.3)		106 (89.1)	48 (92.3)		115 (89.8)	39 (90.7)	
Mucinous carcinoma	8 (5.4)	1 (4.3)		8 (6.7)	1 (1.9)		8 (6.3)	1 (2.3)	
Signet ring cell carcinoma	4 (2.7)	0 (0.0)		4 (3.4)	0 (0.0)		4 (3.1)	0	
Undifferentiated carcinoma	0 (0.0)	4 (17.4)		1 (0.8)	3 (5.8)		1 (0.8)	3 (7.0)	
Tumor grade			0.029 *			0.600			0.854
Low	118 (79.7)	13 (56.5)		93 (78.2)	38 (73.1)		99 (77.3)	32 (74.4)	
High	30 (20.3)	10 (43.5)		26 (21.8)	14 (26.9)		29 (22.7)	11 (25.6)	
Lymphovascular invasion			0.178			0.684			0.454
Absent	71 (48.0)	7 (30.4)		56 (47.1)	22 (42.3)		61 (47.7)	17 (39.5)	
Present	77 (52.0)	16 (69.6)		63 (52.9)	30 (57.7)		67 (52.3)	26 (60.5)	
Predisposing condition			1.000			0.044 *			0.023 *
Absent	127 (85.8)	20 (87.0)		107 (89.9)	40 (76.9)		115 (89.8)	32 (74.4)	
Present	21 (14.2)	3 (13.0)		12 (10.1)	12 (23.1)		13 (10.1)	11 (25.6)	
MSI status			0.690			0.590			0.583
MSS	113 (76.4)	19 (82.6)		90 (75.6)	42 (80.8)		97 (75.8)	35 (81.4)	
MSI-H	35 (23.6)	4 (17.4)		29 (24.4)	10 (19.2)		31 (24.2)	8 (18.6)	
pT classification			0.372			0.585			0.351
pT_is_ − pT_1_	8 (5.4)	1 (4.4)		6 (5.0)	3 (5.8)		7 (5.5)	2 (4.7)	
pT_2_	8 (5.4)	0 (0.0)		6 (5.0)	2 (3.8)		6 (4.7)	2 (4.7)	
pT_3_	47 (31.8)	11 (47.8)		44 (37.0)	14 (26.9)		48 (37.5)	10 (23.3)	
pT_4_	85 (57.4)	11 (47.8)		63 (53.0)	33 (63.5)		67 (52.3)	29 (67.3)	
pN classification ^a^			0.129			0.917			0.852
pN_0_	71 (49.0)	6 (28.6)		53 (45.7)	24 (48.0)		59 (47.2)	18 (43.9)	
pN_1_ + pN_2_	74 (51.0)	15 (71.4)		63 (54.3)	26 (52.0)		66 (52.8)	23 (56.1)	
AJCC staging ^b^			0.215			0.886			0.826
0–I	13 (9.0)	1 (4.8)		9 (7.8)	5 (10.0)		10 (8.0)	4 (9.8)	
II	58 (40.0)	5 (23.8)		44 (37.9)	19 (38.0)		49 (39.2)	14 (34.1)	
III	74 (51.0)	15 (71.4)		63 (54.3)	26 (52.0)		66 (52.8)	23 (56.1)	

^a^ Calculated only 166 cases with available information of lymph node metastasis. ^b^ Calculated only 166 cases with available information of the AJCC stage. * Statistically significant (*p* < 0.05)

**Table 4:** The association between clinicopathologic features and combination of p62 and LC3B expression in patients with SIAC.

	Case *N*	LC3B+/p62^Nu^+, *N* (%)	*p*-Value	LC3B+/p62^Cy^+, *N* (%)	*p*-Value
Gender			0.662		0.970
Male	95	8 (72.7)		8 (66.7)	
Female	76	3 (27.3)		4 (33.3)	
Age			0.017 *		0.056
<60 years	106	2 (18.2)		3 (25.0)	
≥60 years	65	9 (81.8)		9 (75.0)	
Tumor location			0.642		0.430
Proximal (duodenum)	97	5 (45.5)		5 (41.7)	
Distal (jejunum and ileum)	74	6 (54.5)		7 (58.3)	
Histological subtype			<0.001 *		<0.001 *
Adenocarcinoma	154	8 (72.7)		9 (75.0)	
Mucinous carcinoma	9	0 (0.0)		0 (0.0)	
Signet ring cell carcinoma	4	0 (0.0)		0 (0.0)	
Undifferentiated carcinoma	4	3 (27.3)		3 (25.0)	
Tumor grade			0.031 *		0.057
Low	131	5 (45.5)		6 (50.0)	
High	40	6 (54.5)		6 (50.0)	
Lymphovascular invasion			0.342		0.236
Absent	78	3 (27.3)		3 (25.0)	
Present	93	8 (72.7)		9 (75.0)	
Predisposing condition			1.000		1.000
Absent	147	9 (81.8)		10 (83.3)	
Present	24	2 (18.2)		2 (16.7)	
MSI status			1.000		1.000
MSS	132	8 (72.7)		9 (75.0)	
MSI-H	39	3 (27.3)		3 (25.0)	
pT classification			0.492		0.493
pT_is_ − pT_1_	9	0 (0.0)		0 (0.0)	
pT_2_	8	0 (0.0)		0 (0.0)	
pT_3_	58	2 (18.2)		3 (25.0)	
pT_4_	96	9 (81.8)		9 (75.0)	
pN classification ^a^			0.250		0.162
pN_0_	77	2 (22.2)		2 (20.0)	
pN_1_ + pN_2_	89	7 (77.8)		8 (80.0)	
AJCC staging ^b^			0.292		
0–I	14	0 (0.0)		0 (0.0)	0.204
II	63	2 (22.2)		2 (20.0)	
III	89	7 (77.8)		8 (80.0)	

^a^ Calculated only 166 cases with available information of lymph node metastasis. ^b^ Calculated only 166 cases with available information of the AJCC stage. * Statistically significant (*p* < 0.05).

**Table 5:** Univariate analysis of prognostic variables affecting patient survival with SIAC.

Variable	Median Survival (95% CI)	Hazard Ratio (95% CI)	*p*-Value
Age			
<60 years	39.7 (20.5–58.9)	1-	0.116
≥60 years	24.9 (10.5–39.3)	1.35 (0.93–1.95)	
Sex			
Male	36.5 (24.0–49.0)	1-	0.793
Female	28.8 (13.2–44.4)	1.05 (0.72–1.54)	
Location			
Proximal (duodenum)	39.9 (22.7–57.1)	1-	0.153
Distal (jejunum and ileum)	24.5 (15.9–33.2)	1.31 (0.90–1.91)	
Histological subtype			
Adenocarcinoma	36.5 (20.4–52.6)	1-	0.036 *
Mucinous carcinoma	21.0 (5.4–36.6)	1.71 (0.79–3.70)	0.171
Signet ring cell carcinoma	4.2 (NA)	0.95 (0.23–3.85)	0.941
Undifferentiated carcinoma	2.1 (NA)	3.89 (1.42–10.66)	0.008*
Tumor grade			
Low	37.4 (20.0–54.8)	1-	0.172
High	24.5 (16.1–32.9)	1.34 (0.88–2.05)	
Lymphovascular invasion			
Absent	80.8 (40.7–120.9)	1-	<0.001 *
Present	21.1 (14.9–42.7)	2.32 (1.57–3.44)	
Predisposing condition			
Absent	36.5 (21.84–51.16)	1	0.269
Present	18.2 (7.68–28.72)	1.34 (0.80–2.24)	
MSI status			
MSS	28.2 (18.9–37.5)	1	0.029 *
MSI-H	81.7 (6.2–157.2)	0.58 (0.36–0.94)	
pT classification			
pT_is_ − pT_1_	NA	1-	0.025 *
pT_2_	62 (NA)	6.32 (0.71–56.55)	0.099
pT_3_	36.5 (22.3–50.7)	7.96 (1.10–58.16)	0.041 *
pT_4_	22.0 (15.4–28.6)	11.52 (1.60–83.00)	0.015 *
pN classification ^a^			
pN_0_	62.2 (28.4–96.0)	1-	<0.001 *
pN_1_ + pN_2_	22.6 (17.0–28.2)	2.01 (1.36–2.98)	
AJCC staging ^b^			
0-I	NA	1-	0.001 *
II	48.4 (17.9–78.9)	2.76 (0.98–7.76)	0.054
III	22.6 (17.0–28.2)	4.74 (1.72–13.02)	0.003 *
LC3B expression			
Negative	38.5 (23.3–53.7)	1-	0.006 *
Positive	14.7 (11.1–18.3)	1.97 (1.21–3.21)	
p62^Nu^ expression			
Negative	39.7 (23.8–55.6)	1-	0.041 *
Positive	17.8 (10.2–25.4)	1.50 (1.02–2.22)	
p62^Cy^ expression			0.006 *
Negative	41.6 (24.8–58.4)	1	
Positive	15.1 (7.3–22.9)	1.77 (1.18–2.65)	
LC3B+/p62^Nu^+			0.001 *
Absent	48.1 (25.8–70.4)	1	
Present	7.9 (0.0–15.8)	3.32 (1.69–6.53)	
LC3B+/p62^Cy^+			0.002 *
Absent	37.4 (22.9–51.9)	1	
Present	7.9 (0.0–23.5)	2.74 (1.46–5.13)	

^a^ Calculated only 166 cases with available information of lymph node metastasis. ^b^ Calculated only 166 cases with available information of the AJCC stage. * Statistically significant (*p* < 0.05).

**Table 6:** Cox proportional multivariate analysis of the association between prognostic variables and OS in SIAC patients.

Variables	Hazard Ratio (95% CI)	Se (Coef)	z	*p*-Value
MSI-H	0.471 (0.283–0.783)	0.260	−2.899	0.004 *
pT classification (≥pT_2_)	1.534 (1.136–2.071)	0.153	2.792	0.005 *
Nodal metastasis	1.672 (1.112–2.514)	0.208	2.472	0.013 *
LC3B+ expression	1.817 (1.077–3.064)	0.267	2.239	0.025 *
P62^Nu^+ expression	1.334 (0.893–1.991)	0.204	1.409	0.160
P62^Cy^+ expression	0.889 (0.576–1.370)	0.221	−0.535	0.592

* Statistically significant (*p* < 0.05).

## Data Availability

The data that support the findings of this study are available from the corresponding author upon reasonable request.
